# Supplementary material for: Integrated photodynamic Raman theranostic system for cancer diagnosis, treatment, and post-treatment molecular monitoring
Source: Theranostics. 2021 Jan 1;11(4):2006–19. doi: 10.7150/thno.53031 (PMC7778600; doi:10.7150/thno.53031)
Supplement: Supplementary file 1 — Supplementary figures. [file thnov11p2006s1.pdf]

Integrated photodynamic Raman theranostic system for cancer diagnosis, treatment, and post-treatment molecular monitoring

Conor C. Horgan[1,2,3], Mads S. Bergholt[1,2,3†], Anika Nagelkerke[1,2,3#], May Zaw Thin[4], Isaac J. Pence[1,2,3], Ulrike Kauscher[1,2,3], Tammy L. Kalber[4], Daniel J. Stuckey[4], Molly M. Stevens[1,2,3*]

[1]Department of Materials, Imperial College London, London SW7 2AZ, UK.
[2]Department of Bioengineering, Imperial College London, London SW7 2AZ, UK.
[3]Institute of Biomedical Engineering, Imperial College London, London SW7 2AZ, UK.
[4]Centre for Advanced Biomedical Imaging, University College London, London WC1E 6DD, UK.
[†]Current address: Centre for Craniofacial and Regenerative Biology, King's College London, London SE1 9RT, UK.
[#]Current address: University of Groningen, Groningen Research Institute of Pharmacy, Pharmaceutical Analysis, P.O. Box 196, XB20, 9700 AD Groningen, The Netherlands.
[*]Corresponding author: m.stevens@imperial.ac.uk

Supplementary Information

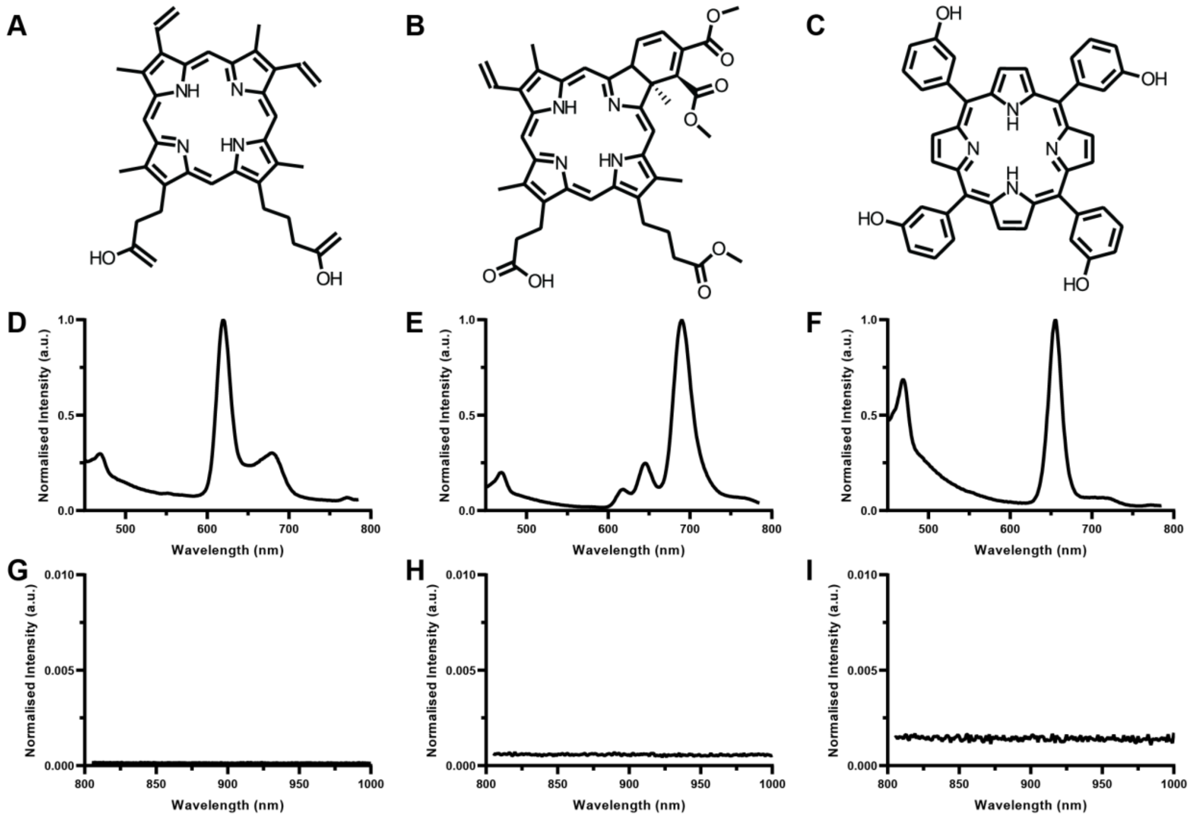

Figure S1 | Photosensitisers for Raman-PDT theranostics. (**A-C**) Chemical structures of photosensitisers investigated for Raman-PDT theranostic system; (**A**) Protoporphyrin IX (PPIX), (**B**) Verteporfin, (**C**) Temoporfin. (**D-F**) Normalised fluorescence emission spectra (ex 405 nm) of (**D**) PPIX, (**E**) Verteporfin, (**F**) Temoporfin. (**G-I**) Normalised fluorescence emission spectra (ex 785 nm) of (**G**) PPIX, (**H**) Verteporfin, (**I**) Temoporfin.

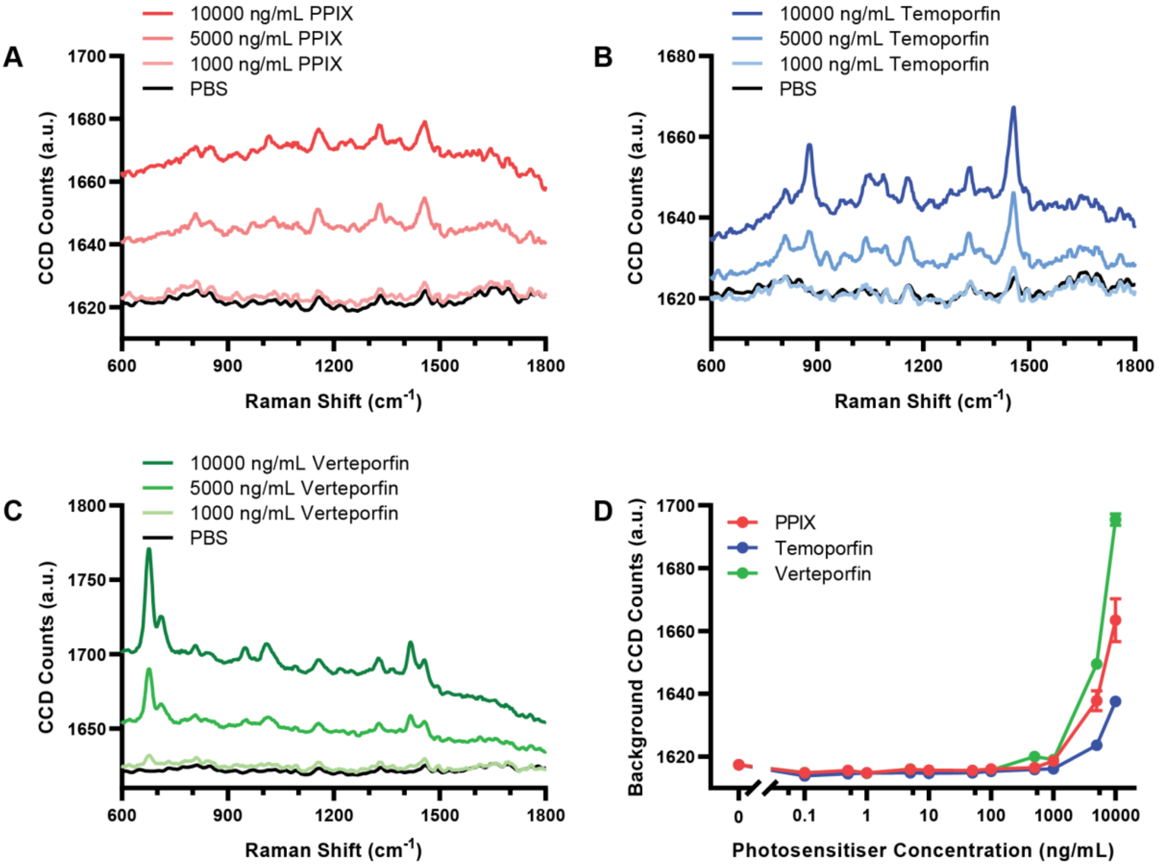

Figure S2 | Raw Raman spectra of photosensitiser solutions. (**A-C**) Raw Raman spectra of (**A**) PPIX, (**B**) Temoporfin, and (**C**) Verteporfin serial dilutions as compared to PBS (n = 5). Major peaks seen in (**B**) and (**C**) correspond to background traces of solvents used in preparation of Temoporfin and Verteporfin solutions. (**D**) Peak fluorescence backgrounds for photosensitizer serial dilutions (mean ± S.D., n = 5).

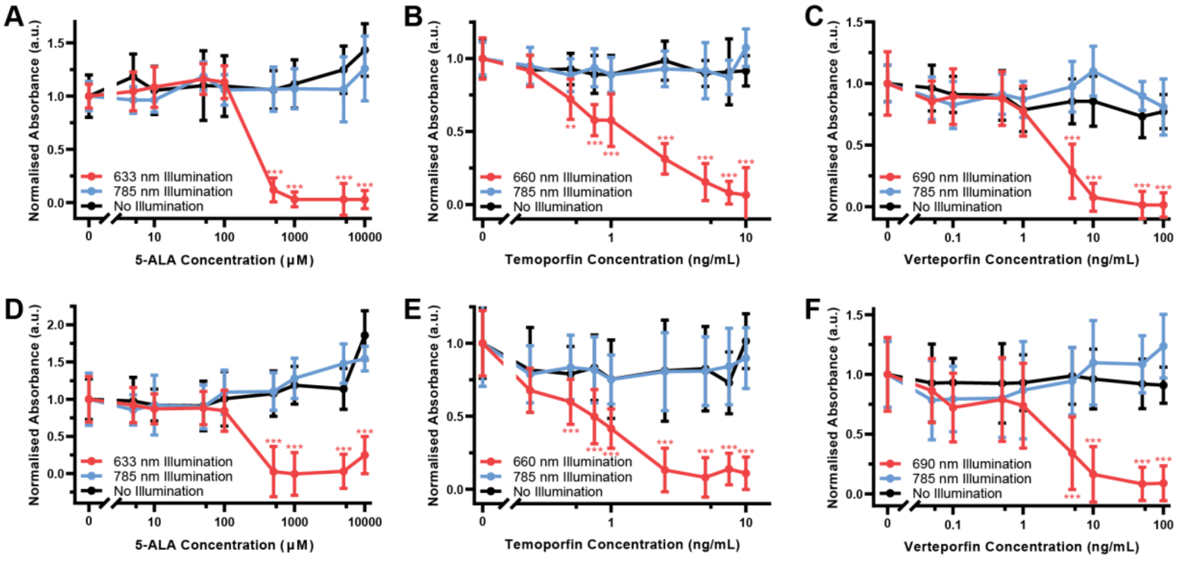

Figure S3 | Photosensitiser cell viability assays. (**A-C**) Cell viability assays of MDA-MB-231 cells incubated with (**A**) 5-ALA, (**B**) Temoporfin, (**C**) Verteporfin. (**D-F**) Cell viability assays of MDA-MB-436 cells incubated with (**D**) 5-ALA, (**E**) Temoporfin, (**F**) Verteporfin. (mean ± S.D., N = 3, n = 6) (Error bars: mean ± STD) (Multiple comparisons *t*-test, Bonferroni post hoc correction, * $P < 0.05$, ** $P < 0.01$, *** $P < 0.001$).

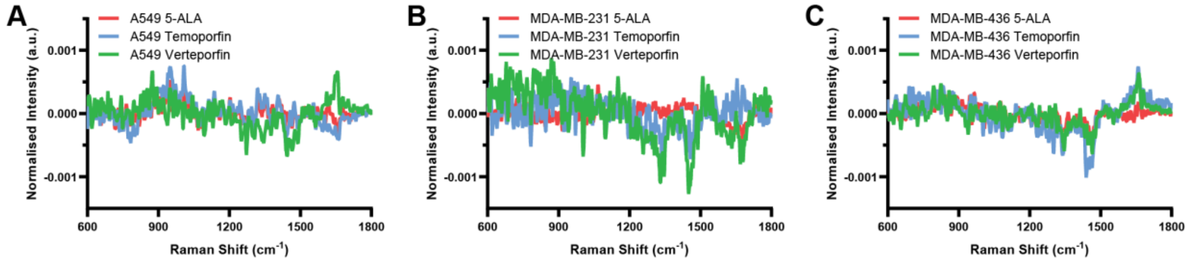

Figure S4 | Raman difference spectra of photosensitiser cells. (**A-C**) Raman difference spectra (10 s integration time) of cells in the presence of different photosensitisers (phenol red-free DMEM (Control), 5-ALA (10000 μM), Verteporfin (100 ng/mL), or Temoporfin (10 ng/mL)), calculated as 'PS Cell – Control Cell' for (**A**) A549 cells, (**B**) MDA-MB-231 cells, and (**C**) MDA-MB-436 cells (N = 10, n = 5).

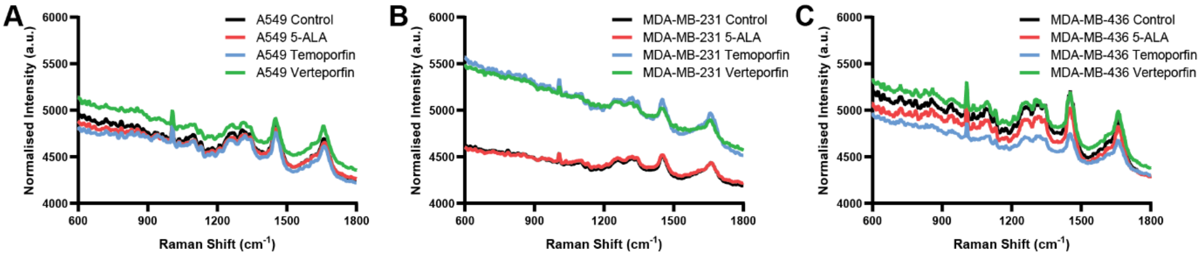

Figure S5 | Raw Raman spectra of photosensitiser cells. (**A-C**) Raw Raman spectral acquisitions (10 s integration time) of (**A**) A549 cells, (**B**) MDA-MB-231 cells, and (**C**) MDA-MB-436 cells in the presence of different photosensitisers (phenol red-free DMEM (Control), 5-ALA (10000 μM), Verteporfin (100 ng/mL), or Temoporfin (10 ng/mL)) (N = 10, n = 5).

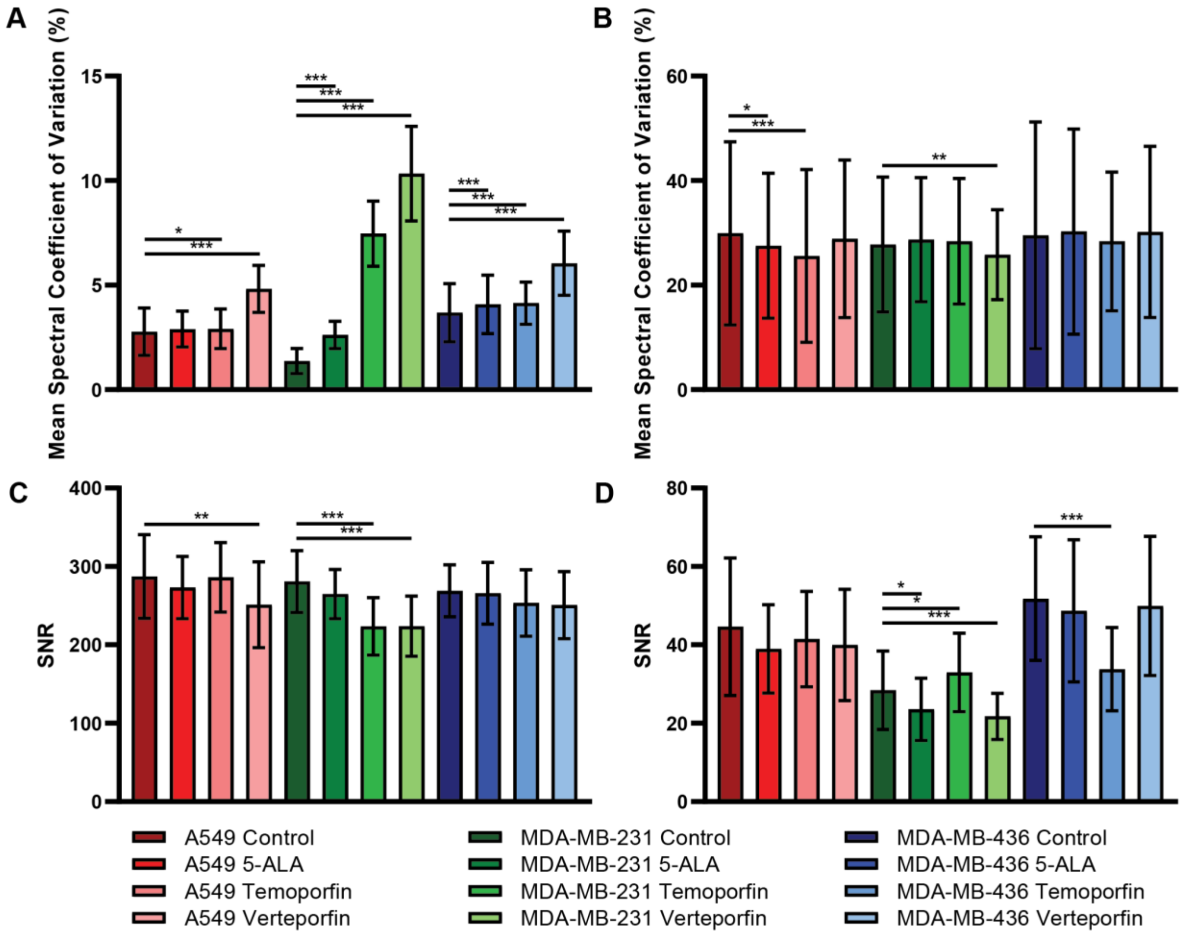

Figure S6 | Mean spectral coefficient of variation and signal-to-noise ratio of photosensitiser cells. (**A-B**) Mean spectral coefficient of variation of (**A**) raw and (**B**) processed Raman photosensitiser cell spectra. (**C-D**) Mean SNR of (**C**) raw and (**D**) processed Raman photosensitiser cell spectra (N = 10, n = 5) (Error bars: mean ± STD) (Two-way analysis of variance (ANOVA), Tukey's honest significant differences (HSD) post hoc correction, * $P < 0.05$, ** $P < 0.01$, *** $P < 0.001$).

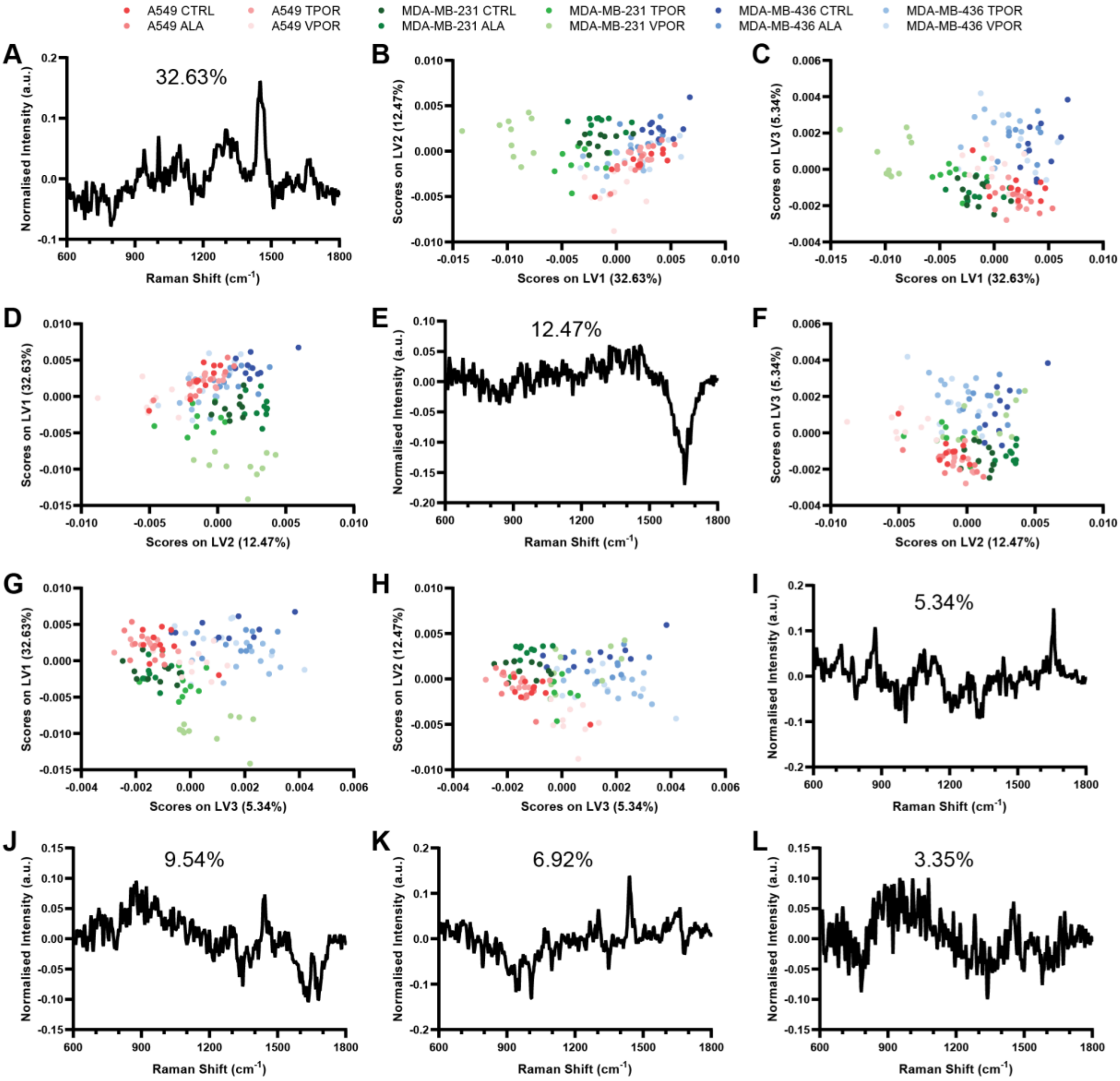

Figure S7 | Photosensitiser cell Raman spectra PLS-DA. (**A-I**) Matrix plot of (**A, E, I**) latent variables 1-3 for PLS-DA of processed Raman spectra performed across the three cell lines, A549, MDA-MB-231, and MDA-MB-436 (blind to the presence or absence of different photosensitisers) (N = 40, n = 5). (**J-L**) PLS-DA latent variables 4-6. Percentages indicate percentage variance explained by each latent variable.

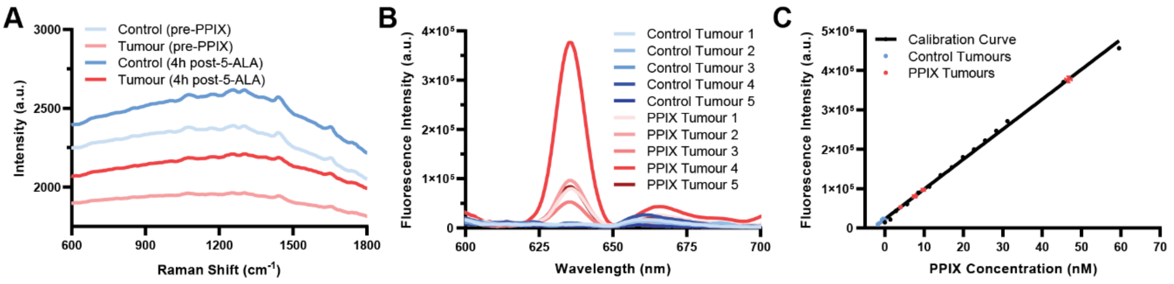

Figure S8 | Confirmation of PPIX uptake in SW1222 tumours *in vivo*. (**A**) Mean raw Raman spectra of control flanks and tumours in mice pre-5-ALA induced PPIX and 4 hours post-5-ALA injection (50 mg/kg) (n = 18-20, N = 5). (**B**) Emission spectra of control tumours and PPIX positive tumours following re-administration of 5-ALA (50 mg/kg) with a 4-hour incubation time 6 days post PDT treatment immediately prior to tumour excision. (**C**) Quantification of PPIX tumour concentration for control and PPIX positive tumours.

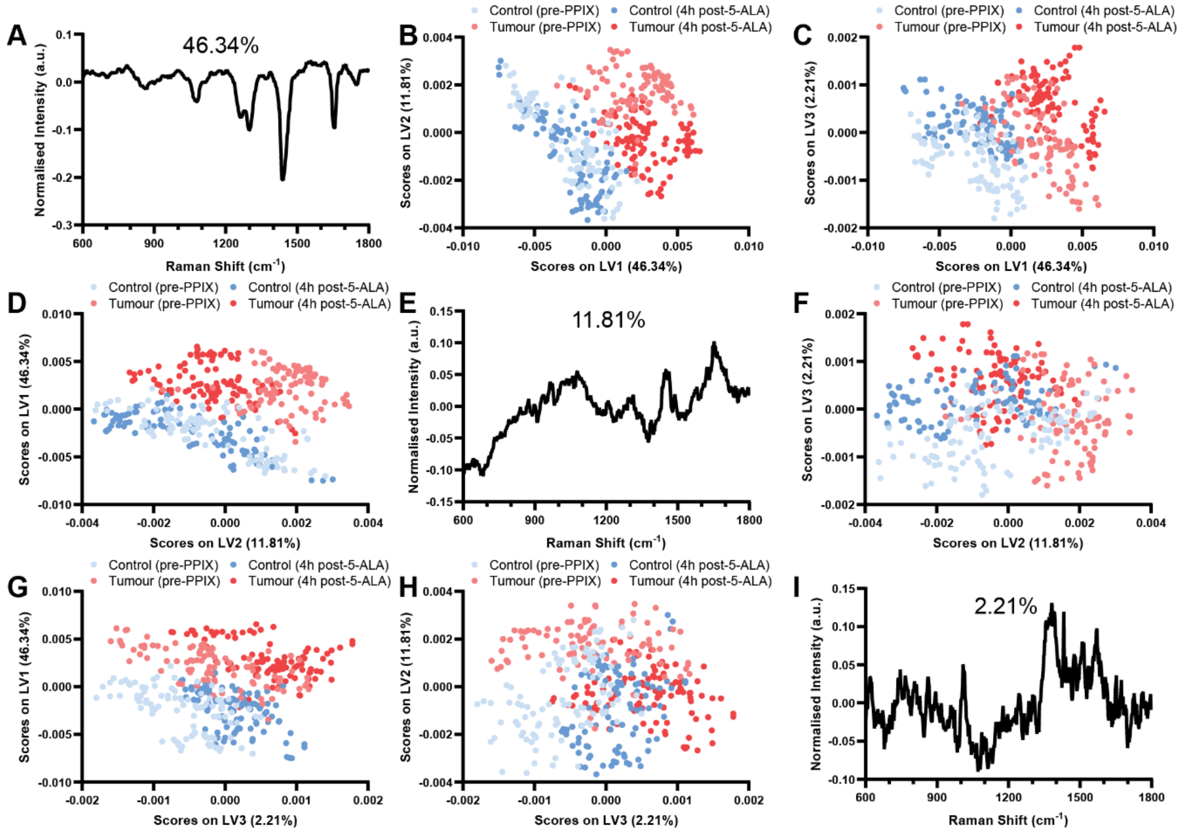

Figure S9 | PPIX+ SW1222 tumours Raman spectra PLS-DA. (**A-I**) Matrix plot of (**A, E, I**) latent variables 1-3 for PLS-DA of processed Raman spectra for control tissue and tumour tissue pre-5-ALA induced PPIX and 4h post 5-ALA injection (50 mg/kg) (n = 18-20, N = 5). Percentages indicate percentage variance explained by each latent variable.
